# Rhizospheric Organic Acids as Biostimulants: Monitoring Feedbacks on Soil Microorganisms and Biochemical Properties

**DOI:** 10.3389/fpls.2020.00633

**Published:** 2020-05-28

**Authors:** Sandra Macias-Benitez, Ana María Garcia-Martinez, Pablo Caballero Jimenez, Juan Miguel Gonzalez, Manuel Tejada Moral, Juan Parrado Rubio

**Affiliations:** ^1^Departamento de Bioquímica y Biología Molecular, Facultad de Farmacia, Universidad de Sevilla, Seville, Spain; ^2^Instituto de Recursos Naturales y Agrobiología de Sevilla, Consejo Superior de Investigaciones Científicas, Seville, Spain; ^3^Departamento de Cristalografía, Mineralogía y Química Agrícola, E.T.S.I.A, Universidad de Sevilla, Seville, Spain

**Keywords:** organic acids, biodiversity, DNA metabarcoding, PGPB, edaphic biostimulation

## Abstract

The biostimulant potential of three different organic acids (OAs) present in the rhizosphere, specifically lactic, oxalic, and citric acids, have been studied. The results showed a rapid and complete metabolism of these three acids with soil microorganisms using them as a source of carbon and energy. Biostimulation was confirmed by soil biochemical studies which showed an increase in enzymatic activities, such as dehydrogenase and phosphatase, lactic and citric acids being those that produced the greatest biostimulation. With regard to microbiota composition, amplicon sequencing of the 16S rRNA gene showed changes in the structure of soil microbial communities. Applying OAs produced a decrease in richness and diversity indices, inducing specific changes in the structure of the microbiological communities. Applying lactic acid induced rapid changes in microbiota composition at both phylum and family taxonomic levels, favoring the proliferation of microorganisms involved in its degradation and soil fertility, such as the genus *Bacillus* and the family Micrococcaceae. Once the lactic acid was degraded, the biodiversity tended to return to similar phyla, but specific distinctive families and genera remained, leaving a pattern of induction of taxa described as plant growth-promoting bacteria (PGPB), such as the *Sinorhizobium* and *Lysobacter* genera, and the Pseudomonaceae family. Similar behavior was found with citric acid, which favored the proliferation and dominance of microorganisms of the Clostridiaceae family, involved in its degradation, as well as microorganisms of both the Micrococcaceae and Pseudomonadaceae families which were found on day 7, leaving a similar pattern of induction as that found after the mineralization of lactic acid. On the other hand, oxalic acid induced long-lasting changes in the bacterial community composition. This was characterized by an increase in the proportion of the Burkholderiales order, which includes microorganisms involved in the degradation of this acid and microorganisms described as PGPB. This study presents evidence supporting the use of OAs as potential soil fertility inducers, due both to their effects in enhancing the dominance of taxa described as PGPB and to their stimulating soil microbial activity.

## Introduction

Soil microorganisms, because they determine soil biochemical properties and soil physicochemical properties such as organic matter, are considered important indicators of soil fertility and productivity (Van Der Heijden et al., 2008; Liang et al., 2018). Plants are able to modify their surroundings by root exudation into the rhizosphere of a large range of compounds that alter soil physical and chemical properties and mediate the interactions between plants and rhizospheric microorganisms (Nihorimbere et al., 2011; Huang et al., 2014). Root exudates are often divided into two classes of compounds: low molecular weight compounds, such as amino acids, organic acids (OAs), sugars, and phenolic compounds, and other secondary metabolites, as well as high molecular weight compounds, such as polysaccharides and proteins (Narasimhan et al., 2003; Bais et al., 2006; Badri and Vivanco, 2009; Huang et al., 2014). Many low molecular weight compounds have been hypothesized to present a functional significance in regulating ecosystem productivity, particularly in the rhizosphere. More specifically, low molecular weight carboxylic acids (LMWOAs) play a significant part in the rhizosphere as essential factors for nutrient acquisition, mineral weathering, and alleviation of anaerobic stress in roots (Blaylock and James, 1994; Zhou et al., 2007; Wang et al., 2015; Adeleke et al., 2017). These LMWOAs (ranging from 46 to 100 Da) are characterized as weak acids that contain a chain of carbon atoms associated with at least one functional acid group (Perminova et al., 2003; Dinh et al., 2017). A number of different OAs have been found in soils (e.g., oxalic, citric, formic, lactic, acetic, etc.; Mimmo et al., 2008; Jiang et al., 2012), the average total content of these acids is estimated as being as much as 10% of the total dissolved organic carbon (Van Hees and Lundstrom, 2000). In a wide range of soils, the concentration of these products in soil solution ranges from 1 to 50 μM (Strobel, 2001), an upper value that is not infrequent (Fox and Comerford, 1990).

The variety of organic compounds released by plants has been postulated as being a key factor in influencing microorganism diversity in the rhizospheres of different plant species (Rovira, 1969; Bowen and Rovira, 1991; Bolton et al., 1992), primarily by selecting organisms able to utilize the carbon source profile produced by the roots (Grayston et al., 1998, 2001). LMWOAs are intimately linked to the carbon cycle and P solubilization and acquisition and are a significant part of the water-soluble fractions of organic molecules released in the rhizosphere not only by root exudates, but also by soil microbial metabolites and organic matter decomposition (Adeleke et al., 2017). OAs are also considered key drivers in bacterial chemotaxis from bulk soil to the rhizosphere (Jones et al., 2003). In this way, a bidirectional relationship is established in which plant roots are able, at least partially, to induce a specific microbial community, their exudates facilitating the formation of a plant-friendly rhizosphere and promoting plant growth-promoting bacteria (PGPB) as well as microbial metabolites that affect soil physical-chemical and biological properties (Swinnen, 1994; Lemanceau et al., 1995; Latour et al., 1996). Therefore, the soil bacterial community structure and diversity could be affected by the application or different LMWOAs.

Soil contains highly diverse microbial communities and it is well-known that the information given by culture-dependent techniques is limited (Nisiotou et al., 2014). At present, the method of preference to survey complex microbial communities is based on 16S rRNA gene amplicon sequencing (Huse et al., 2008; Parlapani et al., 2018). Sample multiplexing is achieved by using sample-specific barcoding tagging which adds great versatility and makes the simultaneous analysis of multiple samples feasible.

The aim of this work is to study whether the treatment of soil with rhizospheric OAs, such as lactic, citric and oxalic acids, may have an effect on both soil physicochemical performance and on the composition of the associated microbial community, and their potential use as soil prebiotics.

Prebiotics are substrates that are selectively metabolized by microorganisms, inducing specific changes in the composition and/or activity of highly diverse microbial communities (Adam et al., 2016). These substrates could be used to stimulate a selected group of beneficial bacteria of interest, enhancing their growth and establishment in certain environments such as soils (Teitelbaum and Walker, 2002; Saulnier et al., 2008). As described above, plants can establish highly specific interactions with soil microorganisms by exudation of rhizospheric compounds, among which OAs seem to play a key role (Teplitski et al., 2000; Jones et al., 2003; Badri et al., 2009; Adeleke et al., 2017). With this in mind, we proposed the addition of OAs in soil, used as a selective source of food capable of stimulating specific bacteria and inducing changes in soil microbiota that could have a beneficial effect on plants.

## Materials and Methods

### Experimental Design

In order to investigate the effects of OAs on biochemical and biological properties of soil, an experimental design was established according to Rodríguez-Morgado et al. (2017). Two hundred and fifty grams of soil were pre-incubated in semi-closed microcosms in the incubation chamber at 30–40% of their water holding capacity, under darkness and at 25°C for 7 days. After this phase, soil samples were mixed with OAs such as: L-lactic (BP, Ph. Eur.) pure, pharma grade (PanReac AppliChem, Barcelona, Spain) (L), oxalic acid 2-hydrate pure (PanReac AppliChem, Barcelona, Spain) (O), and citric acid (Sigma–Aldrich, St. Louis, MO, United States) (Ci).

Each OA was dissolved in distilled water, filtered using 0.2 μm cellulose acetate filters (Sartorius Stedim Biotech), to ensure sterility, and applied to the pre-treated soils at a final concentration of 0.05 mmol/g of dry soil. Soil without OA was used as control (C). Each treatment was replicated three times. Distilled water was added to each soil to reach 60% water-holding capacity.

All treated soil samples were placed in semi-closed microcosms and incubated in the incubation chamber under darkness at 25°C for 0, 1, 5, 7, 12, 21, and 28 days. The samples were removed from the incubation microcosm at each incubation time. Soil samples from 0 time point were collected a few minutes after adding and thoroughly mixing each OA.

For each sample, 10 g of soil were taken, and stored in small glass jars (50 ml) at 4°C for chemical and biochemical analysis, whereas 2 g of soil subsamples were pooled and stored at −80°C in sterile polypropylene centrifuge tubes until DNA extraction.

### Soil Chemical and Biochemical Analysis

The soil used in this study was a Plagic Anthrosol (IUSS Working Group WRB, 2015). The main soil characteristics are shown in Table 1. Soil pH was determined in distilled water with a glass electrode (soil/H_2_O ratio 1:2.5) (MAPA. Métodos Oficiales de Análisis, 1986).

**TABLE 1:** Soil properties (mean ± standard error).

Soil properties	
pH (H_2_O)	7.9 ± 0.2
CO_3_^–2^(g kg^–1^)	204 ± 10
Fine sand (g kg^–1^)	139 ± 33
Coarse sand (g kg^–1^)	385 ± 24
Silt (g kg^–1^)	244 ± 17
Clay (g kg^–1^)	232 ± 9
Clay types	Smectite 64%
	Kaolinite 24%
	Illite 12%
Organic matter (g kg^–1^)	± 0.4
Humic acid-C (mg kg^–1^)	18.2 ± 2.6
Fulvic acid-C (mg kg^–1^)	9.6 ± 1.3
Total N (g kg^–1^)	0.5 ± 0.2
Olsen P (g kg^–1^)	8.6 ± 0.9
Fe (mg kg^–1^)	35.6 ± 3.8
Cu (mg kg^–1^)	9.5 ± 1.2
Mn (mg kg^–1^)	11.1 ± 2.4
Zn (mg kg^–1^)	8.3 ± 1.3
Cd (mg kg^–1^)	6.2 ± 1.5
Pb (mg kg^–1^)	0.39 ± 0.15
Ni (mg kg^–1^)	3.1 ± 0.5
Cr (mg kg^–1^)	5.5 ± 0.7

Organic acids were extracted according to the method described by Bolan et al. (1994). One gram of soil was incubated with 10 ml of extraction buffer (1 N H_2_SO_4_), stirring for 30 min at room temperature. The samples were then centrifuged at 12,000 × *g* for 15 min at 4°C, to remove particles in suspension, and supernatants were filtered through a 0.2 μm regenerated cellulose filter. Concentration of OAs present in each extract were analyzed by RP-HPLC using a LC-4000 JASCO system equipped with a Nova-Pak C18 4 μm (4.6 × 300 mm) reverse phase column (Waters) coupled to a JASCO HPLC UV-475 UV/VIS detector. 20 μl of each sample was injected into the chromatograph and eluted isocratically at a constant flow of 0.7 ml/min using 0.2 M KH_2_PO_4_ + 1.5% methanol adjusted with phosphoric acid at pH 2 as the mobile phase. OAs were detected at a wavelength of 215 nm. Analytes concentrations were determined based on the standard corresponding OAs’ calibration curves. Standards of the three OAs were supplied by Sigma–Aldrich.

Soil dehydrogenase activity was measured by reducing 2-p-iodo-3-nitrophenyl 5-phenyl tetrazolium chloride to iodonitrophenyl formazan (INTF) according to Tabatabai (1994). Product resulting from the reduction was measured at 485 nm using a GeneQuant 1300 spectrophotometer (GE Healthcare Bio-Sciences AB, United States).

Soil acid phosphomonoesterase activity was assayed (Tabatabai, 1994) using p-nitrophenyl phosphate as substrate and modified universal buffer (MUB) substrate buffer (pH 6.5). Then 0.5 M CaCl_2_ and 0.5 M NaOH were added to stop the reaction and to extract the product, p-nitrophenol (PNF), whose concentration was determined photo-metrically at 410 nm.

### Soil DNA Extraction and Illumina MiSeq Sequencing

Total genomic DNA was extracted from soil samples using the DNeasy Power-Soil DNA isolation kit (Qiagen) according to manufacturer’s instructions. DNA was resuspended in a final volume of 100 μl, and a DNA extraction blank was included in each extraction round to check for cross contamination during the DNA extraction process.

For library preparation, the V3–V4 hypervariable regions of the bacterial 16S rRNA gene were amplified using the primer pair Bakt 341F (5’ CCTACG GGN GGC WGC AG 3’)/Bakt 805R (5’ GAC TAC HVG GGTATC TAA TCC 3’) (Herlemann et al., 2011) as the forward and reverse primers with the Illumina-specific sequencing sequences attached to their 5’ ends.

The PCR was performed in 25 μl final volume containing 12.5 μl of Supreme NZYTaq 2x Green Master Mix (NZYTech), 0.5 μM each primer, 2.5 μl of template DNA, and ultrapure water. The PCR program consisted of an initial heating step at 95°C for 5 min, followed by 25 cycles at 95°C for 30 s, 55°C for 30 s, 72°C for 30 s, and a final extension step at 72°C for 10 min.

The barcoding sequences required for multiplexing different libraries in the same sequencing pool were attached in a second PCR round with identical conditions but with only five cycles and with 60°C as the annealing temperature. A negative control containing no DNA was included in order to check for contamination during library preparation.

The correct library size was checked by gel electrophoresis and then the libraries were purified using Mag-Bind RXNPure Plus magnetic beads (Omega Biotek), and pooled in equimolar amounts according to the quantification data provided by the Qubit dsDNA HS Assay (Thermo Fisher Scientific). Finally, the pool was paired-end sequenced in an Illumina MiSeq PE300 platform.

### Analysis of Microbial Community Composition

Sequencing data were processed using Quantitative Insights Into Microbial Ecology (QIIME, version 1.9.0) as described by Caporaso et al. (2010). Raw FASTQ files were demultiplexed, trimmed by CUTADAPT 1.3 (Martin, 2011), merged by FLASH (Magoč and Salzberg, 2011), and quality–filtered and labeled by QIIME 1.9.0 with the following criteria: (i) sequences whose overlap exceeded 30 bp were merged according to their overlap sequence; (ii) primers were matched allowing two nucleotide mismatches, (iii) reads shorter than 300 nucleotides were removed; and (iv) merged reads were quality-filtered at minimum Phred quality score of 20.

All chimeric sequences were identified and removed by the UCHIME algorithm (Edgar et al., 2011) implemented in VSEARCH, using the Greengenes reference database (DeSantis et al., 2006). The sequences were then clustered into operational taxonomic units (OTUs) using the *de novo* approach at the ≥ 100% identity threshold. Singleton OTUs were filtered out, and the representative sequence for each OTU was assigned to a microbial taxon using the RDP classifier (Wang et al., 2007) with a confidence threshold of 97%.

Alpha diversity indices Chao, Good’s coverage, Simpson, Shannon, and phylogenetic diversity were calculated to analyze the complexity of species diversity in each sample.

Operational taxonomic unit data files generated by QIIME were imported in R version 3.5.1 (R Core Team, 2018), so as to further process and visualize results. OTUs counts and taxonomic assignments were merged to a phyloseq object with phyloseq R package (McMurdie and Holmes, 2013).

Rarefaction, relative abundance, and heatmap plots were generated using a combination of Vegan (Oksanen et al., 2018) and ggplot2 (Wickham, 2016) R packages. The principal coordinate analysis (PCoA) using the Weighted-Unifrac distance metric was applied to visualize the microbial community structure in relation to each treatment for each time, and Venn diagrams were generated with VennDiagram (Chen, 2018) R package to depict all possible comparisons of shared, common, and/or unique OTUs, among samples.

The original sequence data have been deposited in the European Nucleotide Archive (ENA) under accession number PRJEB35168.

### Statistical Analysis

The differences in soil chemical and biochemical properties among different treatments were compared using one-way analysis of variance (ANOVA), followed by the least significant difference (LSD) test using agricolae R package (Mendiburu, 2019). All significance levels were set at *p* < 0.05.

## Results

### Influence on pH and Mineralization of Organic Acids on Soil

**FIGURE 1:**
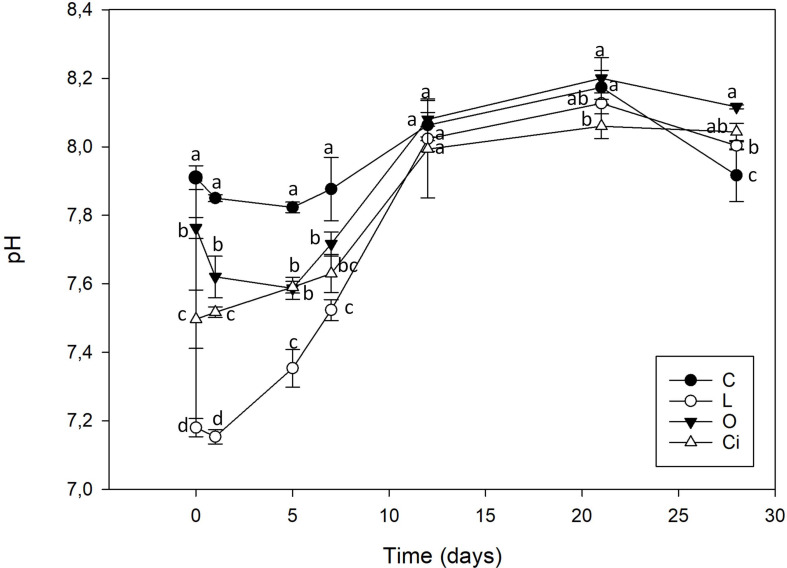
Soil pH after organic acids supplementation. C, control sample; L, lactic acid treated sample; O, oxalic acid treated sample; Ci, citric acid treated sample. Different letters indicate significant difference among treatments (*p* < 0.05).

The addition of OAs induced a decrease in soil pH (Figure 1), whose magnitude was dependent on the OA added. Lactic acid produced the highest decrease in pH, followed by citric acid and oxalic acid, respectively. The initial pH decrease returned to control sample values 12 days after applying the OAs.

This pH value decrease was independent of acid strength. Lactic acid a weak monoprotic acid (pKa: 3.86) was the acid with the greatest capacity to reduce soil pH, from 7.91 to 7.18. Oxalic acid, with two diprotic carboxylic acid groups and citric acid, a triprotic carboxylic acid had a smaller capacity to acidify the soil, reaching to pH 7.61 and 7.50, respectively.

**FIGURE 2:**
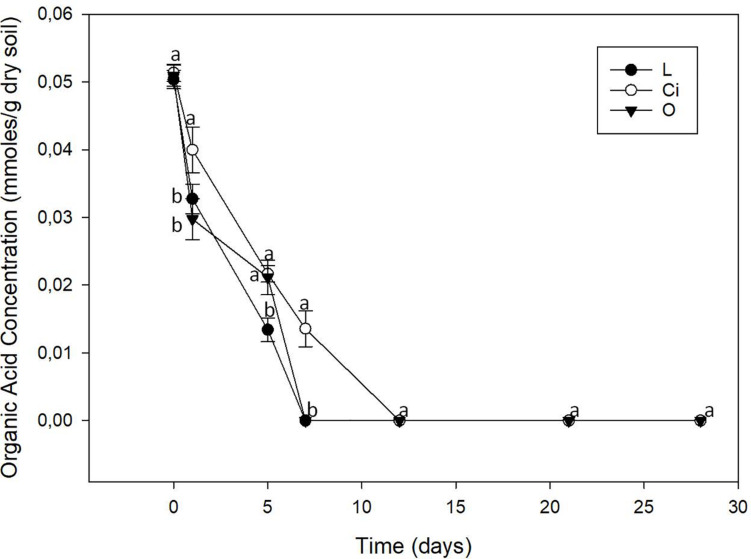
Organic acids content of soil. L, lactic acid treated sample; O, oxalic acid treated sample; Ci, citric acid treated sample. Different letters indicate significant difference among treatments (*p* < 0.05).

The three OAs used were completely mineralized within days of being applied (Figure 2). About 70% of the lactic acid and more than 50% of the citric and oxalic acids had been degraded after 5 days. The lactic and oxalic acids became undetectable after 7 days, whereas the citric acid had a slower mineralization rate. Mineralization of the OAs resulted in a return to control samples pH values.

### Influence of Organic Acids on Soil Enzyme Activities

**FIGURE 3:**
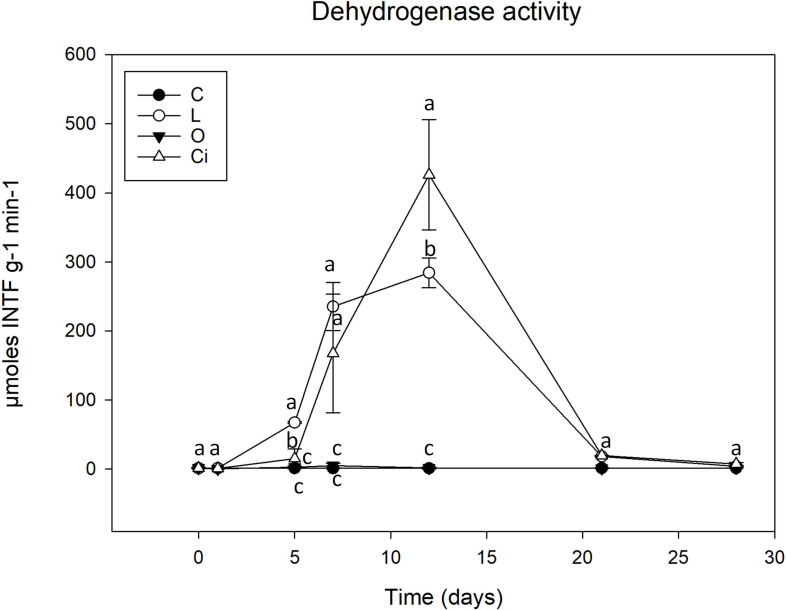
Dehydrogenase activity in soil samples. C, control sample; L, lactic acid treated sample; O, oxalic acid treated sample; Ci, citric acid treated sample. Different letters indicate significant difference among treatments (*p* < 0.05).

**FIGURE 4:**
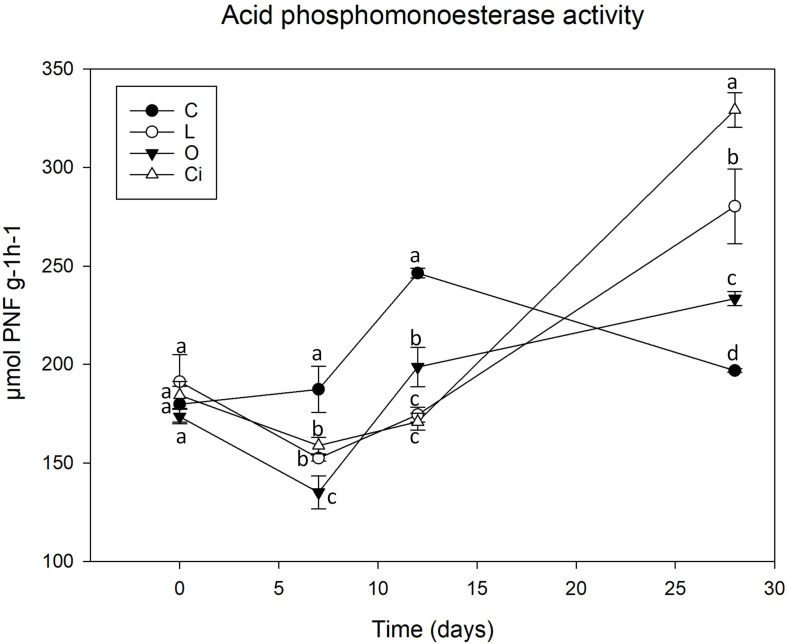
Acid phosphomonoesterase activity in soil samples. C, control sample; L, lactic acid treated sample; O, oxalic acid treated sample; Ci, citric acid treated sample. Different letters indicate significant difference among treatments (*p* < 0.05).

Soil enzymes are the mediators and catalysts of a wide range of soil biological processes and provide an integrative biological assessment of soil functions (Nannipieri et al., 2002). In our present work, two enzymatic activities were measured; dehydrogenase activity, commonly used as an indicator of biological activity in soils, and phosphomonoesterase activity, that has a crucial role in P cycle and correlates with soil phosphate bioavailability (Karaca et al., 2011; Navnage et al., 2018) (Figures 3, 4). Samples treated with lactic and citric acids showed a significant stimulation of dehydrogenase activity with respect to control samples, whereas samples treated with oxalic acid presented an insignificant effect on this activity.

Although both lactic and citric acids produced maximum peaks of dehydrogenase activity around the twelfth experimental day [lactic acid (L), 283.7 μmoles INTFg^–1^min^–1^; citric acid (Ci), 425.7 μmoles INTFg^–1^min^–1^], the samples treated with citric acid resulted in a highest peak of activity which was slightly delayed with respect to the lactic acid-supplemented samples.

From day 12 of the experiment dehydrogenase activity decreased progressively and by the end of the incubation period, the values of treated and control soil samples both presented similar levels (Figure 3).

After 7 days of incubation phosphomonoesterase activity (Figure 4) was decreased due to the addition of the three OAs [L, -18.38%; Ci, -15.65%; oxalic acid (O), -28.55%] 12 (L, -28.57%; Ci, -30.07%; O, -18.69%) with respect to control samples. The results showed that the decrease in phosphomonoesterase activity coincides with the pH drop. At the end of the assay when pH has reverted to the control values and OAs have been completely metabolized phosphomonoesterase activity remained at higher values than controls in all OAs-treated samples (L, + 41.14%; Ci, + 66.15; O, + 17.44%).

### Effects on Soil Bacterial Community Diversity

A total of 85,549 quality bacterial sequences were obtained with a range of 8191–12,534 sequences per sample. To perform the downstream analyses, each sample was normalized to the minimum depth of sequences, which was 8191.

The good coverage indices for all samples were greater than 0.93 (Table 2), and their rarefaction curves (Supplementary Figure S1) approached a level-off indicating that our analysis captured most of the bacterial diversity representative of the samples.

**TABLE 2:** α-Diversity indices of soil samples.

Treatment	Time	Coverage	Observed OTUs	Shannon	Simpson	PD_whole_tree	Chao1
Control	7	0.944	1497	9.2	0.995	67.9	1837
	28	0.946	1480	9.3	0.996	67.5	1793
Lactic	7	0.938	1135	7.4	0.973	56.2	1672
	28	0.945	1212	8.1	0.986	61.8	1620
Oxalic	7	0.940	1317	8.3	0.983	62.9	1723
	28	0.945	1297	8.5	0.983	64.9	1700
Citric	7	0.947	890	5.9	0.884	50.5	1434
	28	0.944	1150	7.8	0.981	59.3	1611

*Coverage, non-parametric coverage estimator; Observed, OTUS observed operational taxonomic units; Shannon, non-parametric Shannon diversity index; Simpson, non-parametric Simpson diversity index; PD_whole_tree, Faith’s Phylogenetic Diversity index; Chao1, richness of the Chao1 estimator.*

Control samples had the highest number of observed OTUs and the highest richness and diversity indices.

Organic acid supplementation produced a decrease in the richness and diversity of the bacterial communities, being more pronounced in the samples treated with citric acid and lactic acid at 7 days. Specifically, on day 7, the sample treated with citric acid showed both the lowest number of OTUs (890) and the lowest values for the diversity indices of Shannon (5.9), Simpson (0.884), and phylogenetic diversity (PD_whole_tree) (50.5) (Table 2).

**FIGURE 5:**
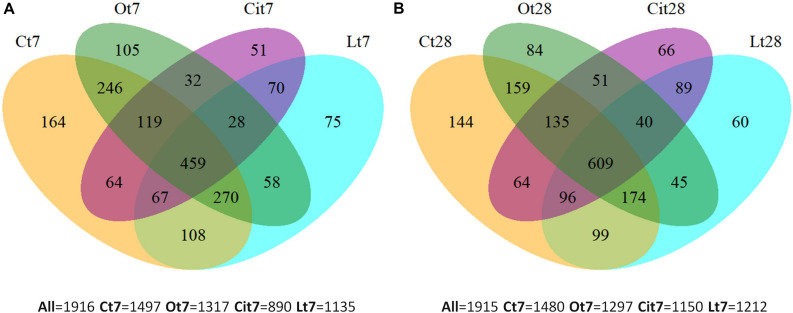
Venn diagrams of unique and shared OTUs among samples at **(A)** 7 and **(B)** 28 days. C, control sample; L, lactic acid treated sample; O, oxalic acid treated sample; Ci, citric acid treated sample.

Venn diagrams (Figure 5) were used to represent the shared and unique OTUs among all the samples after treatment with OAs on both days 7 and 28. Thus, on day 7, all samples shared 459 (23.1%) OTUs, the control sample had 164 (8.56%) unique OTUs and 419 (21.87%) OTUs were specific to the application of the three OAs, of which 28 OTUs were shared among the three OA treatments and 75 (3.85%), 105 (5.48%), and 51 (2.67%) OTUs were unique to the treatments of lactic, oxalic, and citric acid, respectively (Figure 5A). On day 28, all of the samples had 609 (31.8%) OTUs in common, 144 (7.5%) OTUs were found exclusively in the control sample and 435 (22.7%) OTU were detected in samples treated with OAs only, of which 40 OTUs were shared by the three OA treatments, and 60 (3.13%), 84 (4.38%), and 66 (3.44%) OTUs were unique to the samples treated with lactic, oxalic, and citric acids, respectively (Figure 5B).

The Venn diagrams show the existence of OTUs specific to each OA treatment. Microbial communities differed between each other and differed from the control on both days 7 and 28.

**FIGURE 6:**
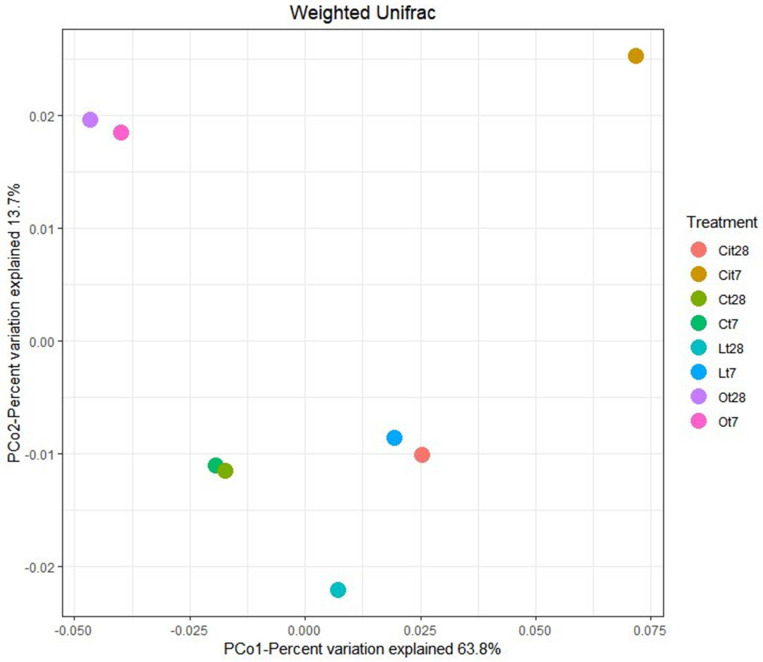
Principal coordinate analysis (PCoA) based on the Weighted Unifrac distance. C, control sample; L, lactic acid treated sample; O, oxalic acid treated sample; Ci, citric acid treated sample. t7: day 7; t8: day 28.

The effect of the application of OAs on the composition of bacterial communities was analyzed by two-dimensional PCoA of weighted UniFrac distance (Figure 6), which explained 63.8% (PCoA1) and 13.7% (PCoA2) of the total variance and confirmed a differentiation between the samples treated with the different OAs and the control samples. On days 7 and 28, samples treated with oxalic acid were grouped in the same cluster, whereas samples treated with lactic and citric acids showed notable differences in the composition between days 7 and 28.

Applying the three OAs studied induced changes in bacterial communities, finding that these communities are different not only from control but also between each other. With regard to OAs, oxalic acid produced a long-lasting change, showing almost identical populations both on days 7 and 28, although samples treated with lactic and citric acids showed different communities on different days during the study.

### Effects on Soil Bacterial Community Composition

**FIGURE 7:**
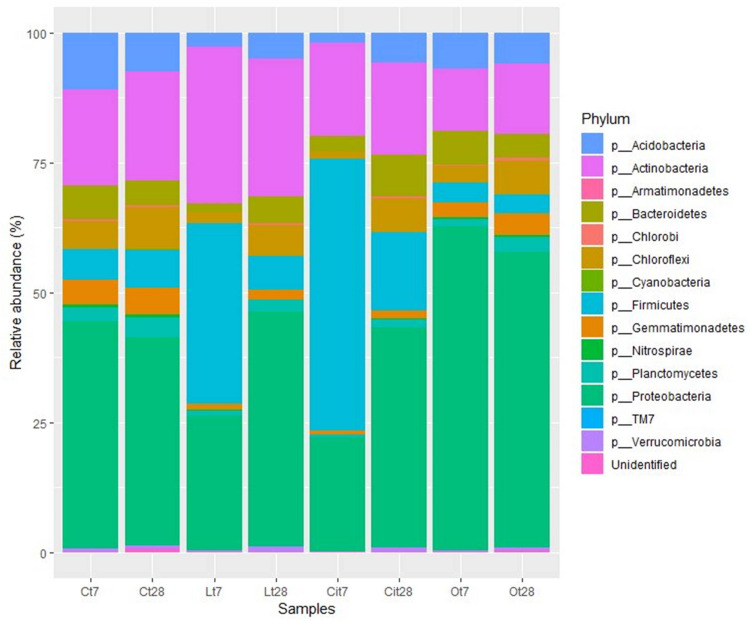
Bacterial community composition at phylum level. Sequences that could not be classified into any known group were designated “Unidentified.” C, control sample; L, lactic acid treated sample; O, oxalic acid treated sample; Ci, citric acid treated sample. t7: day 7; t8: day 28.

Analysis of the taxonomic composition of samples treated with OAs showed that all samples shared a total of 10 phyla. The main phyla for all of them were Proteobacteria, Actinobacteria, Acidobacteria, and Firmicutes, but their abundances differed according to OA supplementation and incubation time (Figure 7).

The soil microbiota in the samples treated with lactic acid on day 7 were dominated by the Firmicutes, Actinobacteria, and Proteobacteria phyla, in this order, which represented 90.7% of the total abundance (Figure 7).

At phylum level, lactic acid produced an increase of 28.7 and 11.1% in the relative abundances of Firmicutes and Actinobacteria, respectively, as well as a decrease of 17.8 and 8.4% in the abundances of Proteobacteria and Acidobacteria.

**FIGURE 8:**
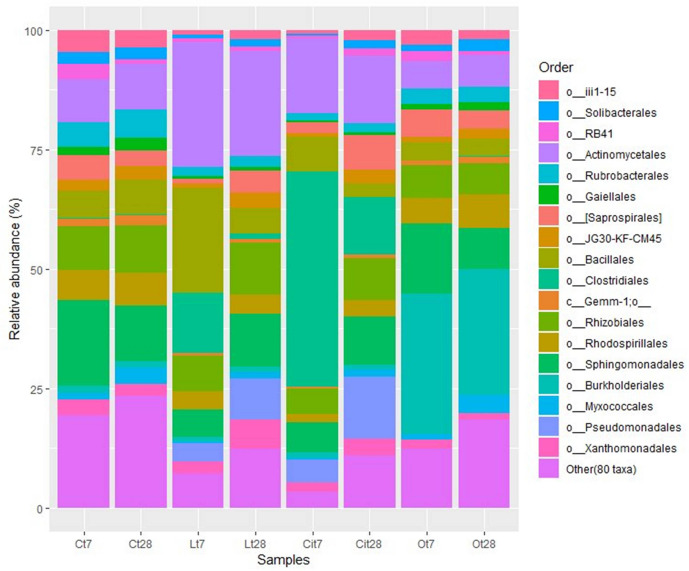
Major bacterial orders. Orders with a total abundance of less 1.0% were collected into “Other (80 taxa).” C, control sample; L, lactic acid treated sample; O, oxalic acid treated sample; Ci, citric acid treated sample. t7: day 7; t8: day 28.

The increase of Firmicutes was due mainly to the increase of 16.1 and 12.6% in the Bacillales and Clostridiales, whose main identified genera were *Bacillus* and *Pelosinus*, respectively (Figure 8 and Supplementary Table S1).

**FIGURE 9:**
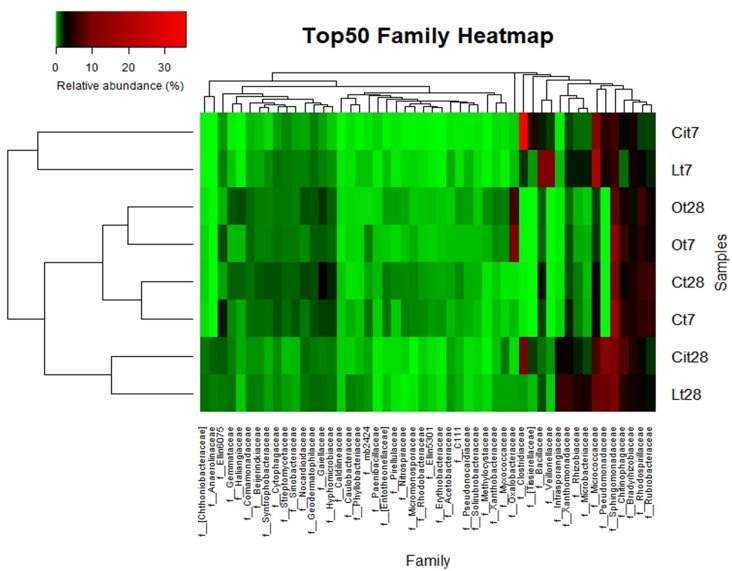
Top 50 most-abundant identified bacterial families. Double hierarchical dendogram of bacterial distribution. Average linkage hierarchical clustering based on Bray–Curtis dissimilarity. C, control sample; L, lactic acid treated sample; O, oxalic acid treated sample; Ci, citric acid treated sample. t7: day 7; t8: day 28.

The increase in the relative abundance of the Actinobacteria phylum corresponded to an increase in the relative abundance of the Actinomycetales order, with an increase of 16.9% in the relative abundance of the Micrococcaceae family in particular (Figures 8, 9).

Within the Proteobacteria phylum, the 17.3% decrease in the relative abundance of the Alphaproteobacteria class should be noted and was paralleled by a decrease in the relative abundance of the Rhizobiales [Lt7 (lactic acid time 7 days), 4.7%; Ct7 (control time 7 days), 9.2%], Rhodospirillales (Lt7, 3.7%; Ct7, 6.3%), and Sphingomonadales (Lt7, 5.8%; Ct7, 17.9%) orders and a 3%increase in the relative abundance of the Gammaproteobacteria, class corresponding to an increase in the relative abundance of the Pseudomonadaceae family (Lt7: 3.8%, Ct7: 0%). On day 28 of incubation, when the OAs had been metabolized, the microbial community of samples treated with lactic acid seemed to restore its bacterial composition with respect to the control at the phylum level. Thus, Proteobacteria became the dominant phylum followed by the Actinobacteria and Firmicutes phyla, with similar relative abundances to those detected in control samples (45.1, 6.5, and 26.1%, respectively).

Looking into changes at lower taxonomical categories during the lactic acid supplemented experiment, we can observe a few differences, mainly in the Proteobacteria and Actinobacteria phyla. Within the Proteobacteria phylum, there was an increase in the relative abundances of the families Rhizobiaceae, Pseudomonadaceae, and Xhantomonadaceae, which presented percentages of relative abundances: 3.6, 12.2, and 4.5%, respectively, higher than control samples.

Within the Actinobacteria phylum the Actinomycetales order maintained a relative abundance similar to that detected on day 7 12.1% higher than that of the control sample, with special stimulation of the Microccocaceae (Lt28, 8.9%; Lt7, 20.4%; Ct28, 3.4%), Microbacteriaceae (Lt28, 4%; Lt7, 2.2%; Ct28, 0.3%), and Intrasporangiaceae (Lt28, 6.8%, Lt7, 0.2%; Ct28, 0.1%) families (Figures 8, 9 and Supplementary Table S1).

Similar to lactic acid-supplemented samples, the soil microbiota in the samples treated with citric acid on day 7 were dominated by the Firmicutes, Proteobacteria, and Actinobacteria phyla, representing 92.4% of the total abundance (Figure 7).

By day 7, citric acid produced an increase of 46.4% in the relative abundances of phylum Firmicutes (Cit7, 52.3%; Ct7, 5.9%), as well as a decrease of 21.8 and 9.1% in the abundances of Proteobacteria (Cit7, 22%; Ct7, 43.8%) and Acidobacteria (Cit7, 1.8%; Ct7, 10.9%) phyla, respectively.

Considering lower taxonomical categories, the increase in Firmicutes corresponded almost entirely to a 38.4% increase in the relative abundance of the family Clostridiaceae. Within the Proteobacteria phylum, the samples treated with citric acid resulted in a decrease of 10.9% (Cit7, 6.3%; Ct7, 17.2%) and an increase of 4.8% (Cit7, 4.8%; Ct7, 0%), of the families Sphingomonadaceae and Pseudomonadaceae, respectively (Figure 9).

Even though on day 7 the Actinobacteria phylum did not present differences respect to the control at phylum level, a marked increase of 9.5% in the relative abundance of the Micrococcaceae family was observed. As in the samples treated with lactic acid, the Acidobacteria phylum decreased its relative abundance.

In parallel to the increase in microbial activity, the presence of citric acid induced a rapid stimulation of specific bacterial populations, as in the case of the Clostridiaceae, Pseudomonadaceae, and Micrococcaceae families (Figure 9).

Like lactic acid, at the end of the incubation period (28 days) when the citric acid had been completely degraded, the composition of the microbial community at the phylum level tended to return to the communities present in the control samples.

On day 28 in the samples treated with citric acid, the Proteobacteria (Cit28, 42.4%; Cit7, 22%; Ct28, 40.2%) phylum had increased its relative abundance by 20.4%, becoming the dominant bacterial phylum, and the Firmicutes (Cit28, 14.9%; Cit7, 52.3%; Ct28, 7.4%) phylum had decreased its relative abundance by 37.4% with respect to day 7. The relative abundance of the Actinobacteria phylum (Cit28, 17.8%; Cit7, 18.1%; Ct28, 21%) remained constant during this period (Figure 7).

By day 28, the Proteobacteria phylum had increased its relative abundance with respect to day 7 and to control samples through the Sphingomonadaceae (Cit28: 9.8%, Cit7: 6.3%, Ct28: 11.1%), Rhizobiaceae (Cit28, 2%; Cit7, 0.4%; Ct28, 0.5%), and Pseudomonadaceae (Cit28, 13%; Cit7, 4.8%; Ct28, 0%) families (Figure 9).

Within the Actinobacteria phylum, by day 28, there was an increase in the relative abundances of the Intransporangiaceae (Cit28, 3%; Cit7, 0%; Ct28, 0.1%) and Microbacteriaceae (Cit28, 1.5%; Cit7, 0.8%; Ct28, 0.3%) families, both with respect to day 7 and to control. Moreover, a decrease of 6.3% in the relative abundance of the family Micrococcaceae (Cit28, 6.7%; Cit7, 13%; Ct28, 3.4%) was observed with respect to day 7.

Within the Firmicutes phylum, there was a 33% decrease in the relative abundance of the order Clostridiales respect to day 7, although it remained 11.9% higher than in the untreated control, samples. This change corresponded to the variations observed in the Clostridiaceae (Cit28, 9.7%; Cit7, 38.4%; Ct28, 0%), Tissierellaceae (Cit28, 1.6%; Cit7, 4.5%; Ct28, 0%), and Veillonellaceae (Cit28, 0.4%; Cit7, 1.5%; Ct28, 0%) families. Within the Firmicutes phylum, the Bacillales (Cit28, 3%; Cit7, 7.4%; Ct28, 7.3%) order also decreased.

The soil microbiota in the samples treated with oxalic acid showed a taxonomic composition differs very much from those of the untreated control and the samples supplemented with lactic and citric acid. After 7 days of treatment, the effect of the oxalic acid resulted in a marked increase of 18.5% in the relative abundance of the Proteobacteria phylum. This phylum was dominant with a representation of 62.3%. This samples presented a decrease in the relative abundances of the Firmicutes (Ot7:3.8%, Ct7: 5.9%), Actinobacteria (Ot7, 11.9%; Ct7, 18.6%), and Acidobacteria (Ot7, 6.9%; Ct7, 10.9%) phyla (Figures 7–9).

That increase within the Proteobacteria phylum was mainly due to the increase in the Burkholderiales (Ct7, 1.6%; Ot7, 29.4%/increase of 27.8%) order (Figures 7, 8).

Unlike in the cases of lactic and citric acids, the addition of oxalic acid produced a long-lasting modification of soil microbiota. After 28 days of incubation, when the oxalic acid had been completely mineralized, the soil bacterial community was highly similar to that at day 7.

On days 7 and 28 of incubation, the samples treated with oxalic acid were dominated by the Burkholderiales order, representing, in both cases, more than 25% of the communities in these samples, but this order only represented less than 1% in the other treatments. Within this order, the Oxalobacteraceae family (Ot7, 10.2%; Ot28, 6.9%, in the rest of treatments around 0.2%) was dominant (Figures 7–9 and Supplementary Table S1).

## Discussion

Organic acids are one of the main components of rhizospheric exudates. They are strongly involved in the solubilization and acquisition of essential nutrients such as P are used as carbon and energy sources by soil microorganisms, and act as key drivers in rhizospheric bacterial chemotaxis (Jones et al., 2003). In short, OAs are associated with different processes that seem to play a key role both in soil fertility and in the development and growth of plants.

The present study focuses on monitoring the effects produced by the addition to the soil of three specific OAs present in rhizospheric exudates and on their characterization as edaphological biostimulants and soil prebiotics. In order to achieve the proposed objectives, different chemical and biochemical analyses of the soil were carried out and the composition of the soil microbiota was evaluated using a metabarcoding approach. Results have important implications for both soil microbiological process and plant growth.

### OAs Fate, Soil Chemical, and Biochemical Properties

The concentration of OAs used in this study falls within the range described in soil extracts (Strobel, 2001). Nevertheless, the concentration of these products varies greatly and by different abiotic and biotic stresses, such as physiological stress, nutrient stress, and some physical changes in soil also have an influence (Chen and Liao, 2016; Dinh et al., 2017; Jiang et al., 2017). Moreover, it is suggested that plant roots’ production of OAs vary among plant species and is influenced considerably by the plant’s developmental stage (Dinh et al., 2017).

The ability to lower soil pH is specific to each type of OA and this behavior may be explained due to a differential chemical interaction between the acids and other soil constituents. The participation of citric and oxalic acids dissolving soil carbonates has been described to buffer pH (Duquène et al., 2008). Furthermore, previous reports mentioned that the application of citric and oxalic acids at low concentrations increased soil pH (Duquène et al., 2008). Therefore, citric and oxalic acids would be participating in the dissolution of carbonates, precipitating in the form of oxalate and calcium citrate, releasing CO_2_ and H_2_O, and increasing bicarbonate levels, which would greatly buffer the potential decrease of soil pH.

The return of pH values to those of control samples was in parallel with the mineralization of the OAs. The three OAs were completely mineralized, probably due to their consumption by soil microflora, soil microorganisms using them as a source of carbon and energy (Rodríguez-Morgado et al., 2017).

With regard to enzyme activities, changes in dehydrogenase activity reflect soil status (Doi and Ranamukhaarachchi, 2009) and have been proposed as a good indicator of soil microbial activity. It is, therefore, widely used as an integrated measure of soil quality (Maliszewska and Smreczak, 2003). As described, dehydrogenase activity was stimulated after treatment with lactic and citric acid (Figure 3). The stimulation would justify the degradation of the citric and lactic acids by soil microorganisms, the citric acid being the richest carbon source, and producing a greater induction of microbial metabolism.

On the other hand, oxalic acid, despite being completely metabolized, did not produce a significant increase in dehydrogenase activity. This behavior could be due to the fact that oxalic acid would be a carbon source used by a small fraction of the microbial community of oxalotrophic bacteria, including Burkholderiales order (Sahin et al., 2009; Palmieri et al., 2019). As a result, oxalic acid could be favoring a small group of specific bacteria that could be metabolizing it without increasing its biomass during the process, and consequently without producing an increase in dehydrogenase activity (Landi et al., 2006).

The changes in phosphomonoesterase activity found in our study may be associated to P bioavailability. Increase of P bioavailability in soils is linked to an inhibition of the enzymatic activity due to the fact that production and excretion of hydrolytic enzymes is stimulated by several signals that convey information about the utility of these enzymes during nutrient-limited growth conditions (Cezairliyan and Ausubel, 2017). Accordingly, previous works described that LMWOAs were able to increase the bioavailability of P in soils during the initial stage of incubation, increasing P bioavailability for microbial growth (Palomo et al., 2006; Clarholm et al., 2015). So the higher P bioavailability due to OAs treatment would explain the inhibition of phosphatase enzymes, as P requirements for growth are being satisfied by its availability in soils. The ability of OAs to mobilize P is already known, so it is reported that low molecular weight OAs such as citric and oxalic acids are among the most commonly produced root and microbial exudates which affect rhizosphere P availability (Clarholm et al., 2015; Menezes-Blackburn et al., 2016). These two OAs induce a higher increase in P mobilization than other OAs (Gerke et al., 2000; Giles et al., 2012) through a different mechanism such as rhizosphere acidification which increases P availability and also forms stable complexes with metal cations and competes with P for adsorption sites on soil colloids (Menezes-Blackburn et al., 2016).

During the experimental incubation, the OAs’ mineralization led to a progressive decrease in the availability of P and the microorganisms reactivated their own production of phosphatases to satisfy their current demand of P (Heitkötter et al., 2017) as shown by our results (Figure 4). Thus, these OAs may contribute to the release of bioavailable P while simultaneously stimulating microbial growth and P sequestration for microbial biomass.

Although oxalic acid did not induce an increase in microbial metabolic activity, the increase in phosphomonoesterase activity detected at the end of the assay could be explained by the induction of specific taxa, in particular the Burkholderiales order and the Oxalobacteraceae family described as effective P solubilizers (Silva et al., 2017).

### OAs and Soil Microbiota

One of the main objectives of this study was to verify how three specific OAs affect the soil microbiota. To this end, bacterial abundance and diversity in soil were assessed using the culture-independent analysis by 16S amplicon sequencing using the Illumina MiSeq platform, the analysis enabling observation of how applying the three OAs tended to produce a decrease in both the diversity and the richness of the microbiota (Table 2). This could be explained by inducing that these supplementary carbon sources are stimulating the proliferation of specific, possibly specialized microorganisms that could be involved in their mineralization—these microorganisms belonging to certain taxonomic categories that would dominate the population.

In agreement with this hypothesis, previous studies have already shown that the application of oxalic and citric acids produced changes in the soil microbial community which were more marked than those produced by the addition of other carbon sources such as glucose and glycine, justified by the fact that these OAs could only be mineralized by certain specialized microorganisms while glucose and glycine could be used by a large part of the microorganisms present in the soil (Falchini et al., 2003; Landi et al., 2006; Eilers et al., 2010). Shi et al. (2011) using DGGE and PhyloChip analyses also showed that OAs stimulated the soil microbial community and produced changes in its composition that were considerably greater than those produced by sugars only. But in contrast to our results, they observed that the OAs produced an increase in diversity and richness indices, something that could be explained by the fact that they used mixtures of OAs with sugars instead of pure OAs.

Supporting our previous explanation, we were also able to verify how applying OAs promoted the proliferation of unique OTUs that were not present in untreated samples. Some of these were found in all of the samples treated with the three LMWOAs, while others were specific to each one, something which evident both on days 7 and 28 of incubation (Figure 5). In Venn diagrams, we could also observe the existence of specific OTUs in the control samples, such taxa would be responding negatively to the application of OAs. This negative impact may result from the direct inhibition of microorganisms due to the presence of OAs. Thus these taxa were outcompeted by the rapid growth of other microorganisms (Paterson et al., 2007; Shi et al., 2011). Moreover, PCoA ordination analysis showed large differences between bacterial community structure with the samples treated with the three OAs and the untreated samples throughout the assay (Figure 6).

At high taxonomic rank composition, Proteobacteria, Actinobacteria, Acidobacteria, and Firmicutes were the predominant phyla in all samples (Figure 6). This is to be expected, since they have been described as some of the phyla that recapitulate most of the diversity of contrasting soil biomes (Fierer and Jackson, 2006; Bulgarelli et al., 2013). But the three OAs induced changes in the representation of these phyla compared with control samples. Lactic and citric acids produced a reversible increase in the relative abundances of the Actinobacteria and Firmicutes phyla, and of the Firmicutes phylum, respectively, returning to initial values at the end of the assay, while oxalic acid produced an increase in the relative abundance of the Proteobacteria phylum that remained unchanged throughout the entire assay.

By delving into the taxonomic categories, we were able to verify how at 7 days of incubation, the stimulation of the *Bacillus*, *Pelosinus*, and *Sinorhizobium* genera in the samples treated with lactic acid stood out, and in the samples treated with citric acid that of the Clostridiaceae family. Interestingly, this effect was simultaneous, with high values of stimulation of the dehydrogenase activity of the samples treated with lactic and citric acids. A common induction of the Micrococcaceae and Pseudomonadaceae families after lactic and citric application was observed (Figure 9 and Supplementary Table S1).

While the *Bacillus* and *Pelosinus* genera would be involved in the degradation of lactic acid. These genera have been characterized by proliferating in acidic environments (Hansel et al., 2008) and by the use of lactate as a source of C (Mosher et al., 2012; Beller et al., 2013); the Clostridiaceae family—which would account for almost 50% of the relative abundance of the bacterial population of samples treated with citric acid—would probably be directly involved in the degradation of this acid (Antranikian and Gottschalk, 1982). On the other hand, the application of both citric and lactic acids would also be promoting the proliferation of beneficial bacteria of agricultural importance by stimulating microorganisms belonging to the *Bacillus* and *Sinorhizobium* genera in the case of lactic acid, as well as the Micrococcaceae and Pseudomonadaceae families in the case of both acids, since PGPB microorganisms have been described within these taxa due to their involvement both in biocontrol, as in the production of ammonium, indolacetic acid, atmospheric N_2_ fixation, P solubilization, etc. (Kumar et al., 2012; Souza et al., 2015; Karličić et al., 2016; Turan et al., 2016; Radhakrishnan et al., 2017).

In a previous study with lactic acid applied to soil, DGGE analyses showed microbial community changes similar to those observed in this study by barcoded, amplicon sequencing data. Specifically, DGGE bands related to the *Pseudomonas*, *Bacillus*, and *Rhizobium* genera were observed (Rodríguez-Morgado et al., 2017).

On day 28, once the lactic and citric acids had already been completely mineralized (Figure 2), biodiversity tended to return to the initial levels when assessing data on phyla (Figure 7), but an induction pattern of PGPB microorganisms, such as the genera *Sinorhizobium* and *Lysobacter*, and the Pseudomonaceae family was observed and maintained at the end of the experiment—suggesting an important involvement of this LMWOAs in soil fertility (Figure 9 and Supplementary Table S1) (Islam, 2011; Gupta et al., 2015; Prasad et al., 2015; Souza et al., 2015; Sharma et al., 2017).

Applying oxalic acid to soil produced long-lasting effects in the taxonomic composition of soil bacterial communities, inducing the proliferation of specific microorganisms involved in its mineralization, despite its not producing a remarkable induction of its metabolic activity. These observations are in agreement with the findings of Landi et al. (2006), who observed that oxalic acid influenced the bacterial communities of the rhizosphere although net soil ATP remained relatively stable, suggesting insignificant changes in bacterial biomass. These small changes in biomass could be the result of the activation of only small fractions of the community, specifically the oxalotrophs (Messini and Favilli, 1990; Palmieri et al., 2019) which represent those bacteria able to mineralize oxalic acid. Other authors have also shown changes in soil bacterial communities induced by oxalic acid (Falchini et al., 2003; Yuan et al., 2018) in spite of a limited modification of the overall soil bacterial biomass.

The Burkholderiales order dominated the samples treated with oxalic acid from day 7 onward. Within this order, the detection of microorganisms belonging to the family Oxalobacteraceae should be highlighted (Figures 8, 9). These taxa could be involved in the mineralization of oxalic acid and it could also play a potentially important role in soil fertility because this order includes numerous representatives such as effective P solubilizers and PGPB (Baldani et al., 2014; Souza et al., 2015; Silva et al., 2017).

## Conclusion

In summary, we conducted an exhaustive study of the influence of OAs common in the rhizospheres, which provided fundamental knowledge for studying their potential use as soil prebiotics. The stimulating effect of OAs on microbial activity in soils is illustrated by the induction of specific bacterial groups with known PGPB roles in soils. In this sense, our study highlights the potential use of rhizospheric OAs as biostimulants to enhance crop yield in a sustainable way.

## Data Availability Statement

The datasets generated for this study can be found in The European Nucleotide Archive (ENA) under accession number PRJEB35168.

## Author Contributions

SM-B and JP designed the study. SM-B, JP, and PC performed research. SM-B, AG-M, PC, JG, MT, and JP analyzed data. SM-B, AG-M, and JP wrote the article.

## Conflict of Interest

The authors declare that the research was conducted in the absence of any commercial or financial relationships that could be construed as a potential conflict of interest.
