# Supplementary figures and images for: Rhizospheric Organic Acids as Biostimulants: Monitoring Feedbacks on Soil Microorganisms and Biochemical Properties
Source: Front Plant Sci. 2020 May 28;11:633. doi: 10.3389/fpls.2020.00633 (PMC7270406; doi:10.3389/fpls.2020.00633)

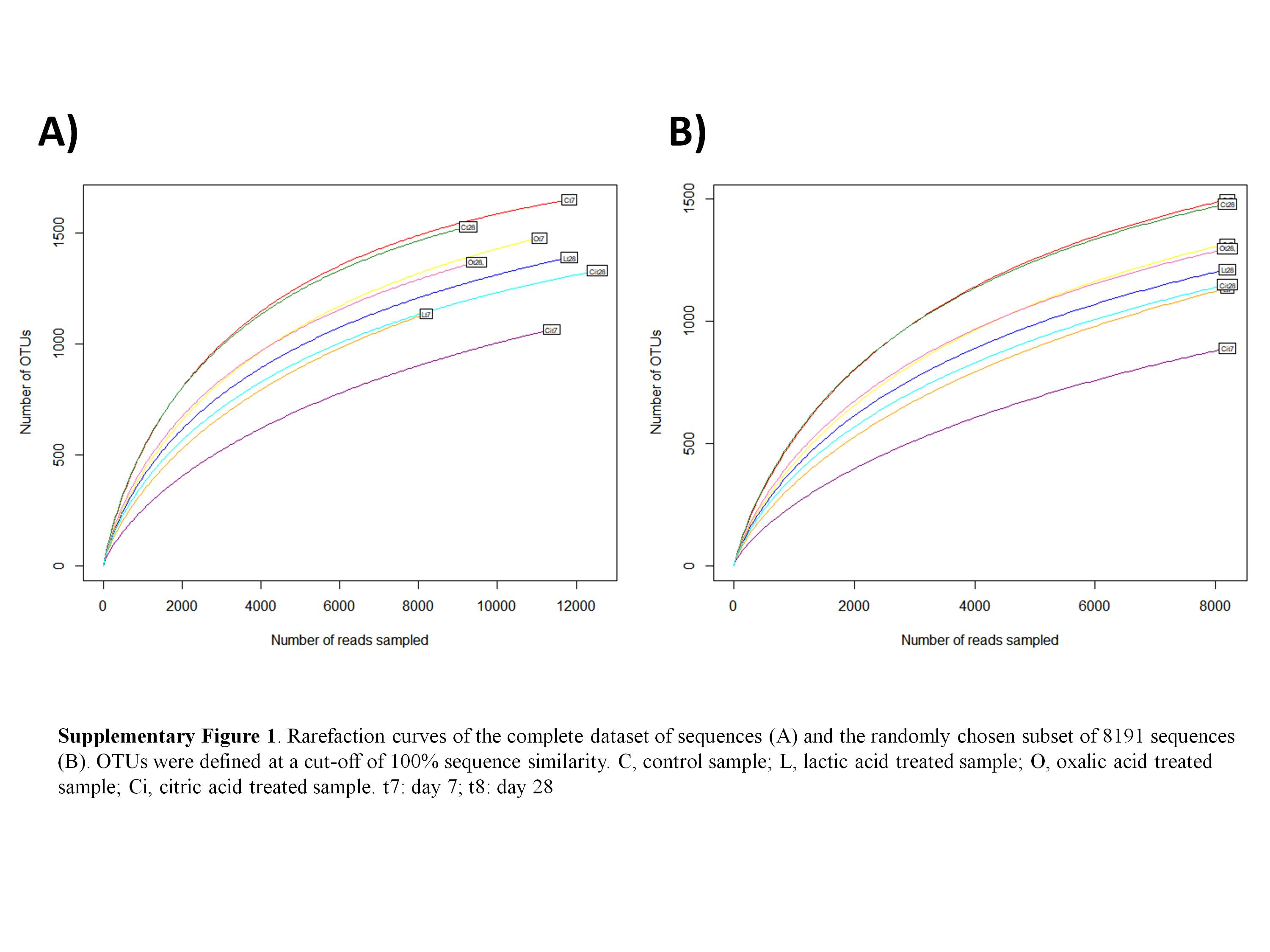

Supplement: Supplementary file 1 [file Image_1.JPEG]
